# Supplementary material for: Regulation of FT splicing by an endogenous cue in temperate grasses
Source: Nat Commun. 2017 Feb 1;8:14320. doi: 10.1038/ncomms14320 (PMC5296679; doi:10.1038/ncomms14320)
Supplement: Supplementary Information — Supplementary Figures and Supplementary Tables [file ncomms14320-s1.pdf]

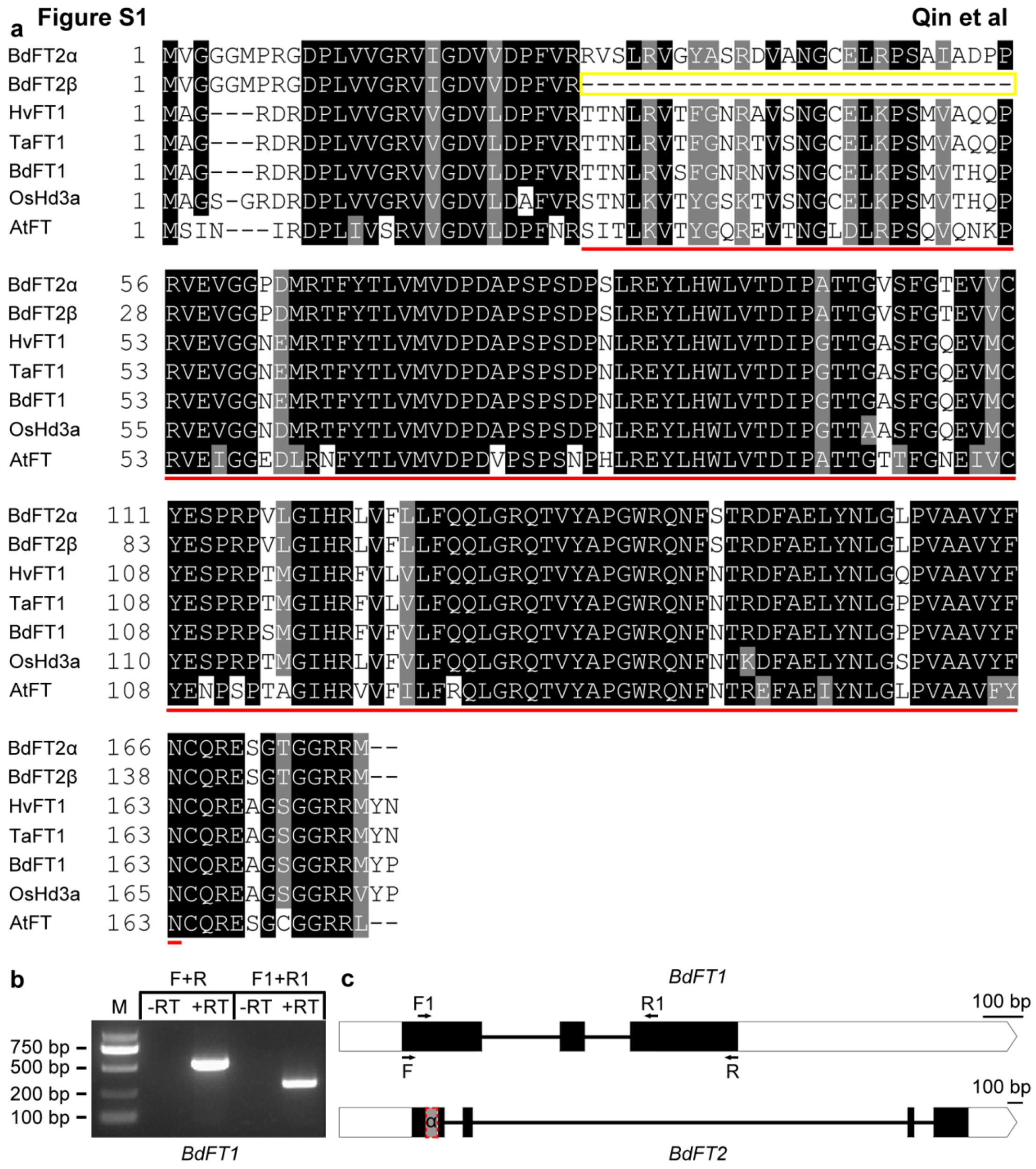

Supplemental Figure 1 ***FT2*** **but not** ***FT1*** **in** ***B. distachyon*** **is subject to AS**

(a) Protein sequence alignment of FT proteins in different plants. Amino acids of PEBP domain are marked by red line. The missing amino acids of FT2β are highlighted by a yellow frame.

(b) Detection of *FT1* transcripts by RT–PCR.

(c) Schematic representation of gene structures of *FT1* and *FT2*. Gene exons and introns are shown by rectangles and lines, respectively. *FT2α* and *FT2β* are differentiated by dotted rectangles.

Figure S2 **Qin et al**

a

FT2α

Concentration (mol/μL)	1e-23	1e-22	1e-21	1e-20	1e-19	1e-18
**Replicate 1**						
	32.0	27.4	23.1	21.3	17.0	14.5
CT values	32.1	27.4	23.4	21.8	16.7	14.5
	30.9	27.4	22.7	21.5	16.6	14.5
Mean CT values	31.7	27.4	23.1	21.6	16.8	14.5
**Replicate 2**						
	31.5	27.5	24.9	21.1	16.7	14.6
CT values	31.3	27.4	23.9	21.4	16.6	14.6
	31.5	28.0	23.1	21.4	16.6	14.6
Mean CT values	31.4	27.6	24.0	21.3	16.6	14.6
**Replicate 3**						
	31.6	28.6	24.7	20.3	17.4	15.2
CT values	31.4	28.6	24.4	20.7	17.2	15.1
	31.8	29.1	23.9	20.5	17.3	15.1
Mean CT values	31.6	28.8	24.3	20.5	17.3	15.1

FT2β

Concentration (mol/μL)	1e-23	1e-22	1e-21	1e-20	1e-19	1e-18
**Replicate 1**						
	30.1	27.1	23.1	20.3	17.1	14.6
CT values	30.3	27.4	23.1	20.3	17.0	14.7
	30.2	27.7	23.0	20.3	17.0	14.7
Mean CT values	30.2	27.4	23.1	20.3	17.0	14.7
**Replicate 2**						
	29.8	26.3	23.1	19.1	16.1	14.3
CT values	29.3	26.4	23.1	19.1	16.1	14.3
	28.5	26.7	23.0	19.2	16.1	14.3
Mean CT values	29.2	26.5	23.1	19.1	16.1	14.3
**Replicate 3**						
	29.2	26.2	23.1	19.2	16.1	14.3
CT values	29.5	26.5	22.9	19.1	16.2	14.3
	29.2	26.7	23.0	19.1	16.2	14.3
Mean CT values	29.3	26.5	23.0	19.1	16.1	14.3

UBC18

Concentration (mol/μL)	1e-22	1e-21	1e-20	1e-19	1e-18
**Replicate 1**					
	27.9	24.4	21.0	18.1	15.3
CT values	27.7	24.4	21.0	18.1	15.2
	27.9	24.4	21.1	18.0	15.2
Mean CT values	27.8	24.4	21.1	18.1	15.2
**Replicate 2**					
	28.4	24.4	21.7	18.1	15.4
CT values	28.3	24.6	21.8	18.1	15.3
	28.3	24.4	21.8	18.2	15.4
Mean CT values	28.4	24.5	21.8	18.1	15.4
**Replicate 3**					
	28.2	24.8	21.1	18.0	15.3
CT values	28.0	24.6	21.4	18.0	15.3
	28.2	24.8	21.4	18.1	15.3
Mean CT values	28.1	24.7	21.3	18.1	15.3

b

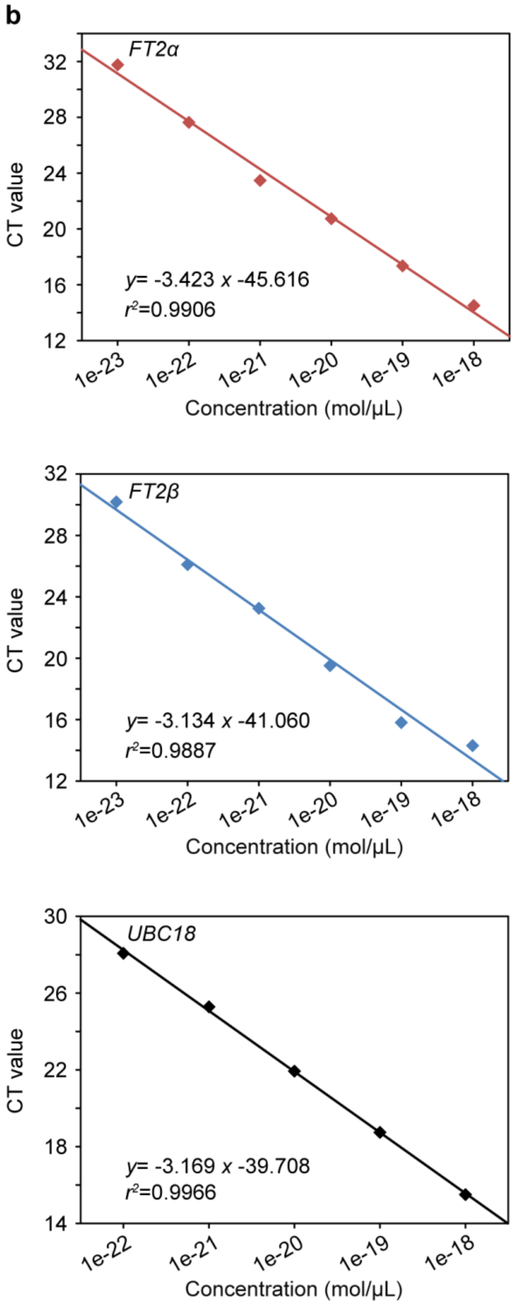

Supplemental Figure 2 Standard curve for quantification of *FT2α, FT2β* and *UBC18* transcripts

(a) CT values in qRT-PCR analyses using dilutions of *FT2α*, *FT2β* and *UBC18* template DNA in three independent experiments.

(b) Standard curves for quantification of *FT2α*, *FT2β* and *UBC18* transcripts, respectively. Each threshold cycle at y-axis is the average CT value from three replicates shown in (a).

Figure S3 **Qin et al**

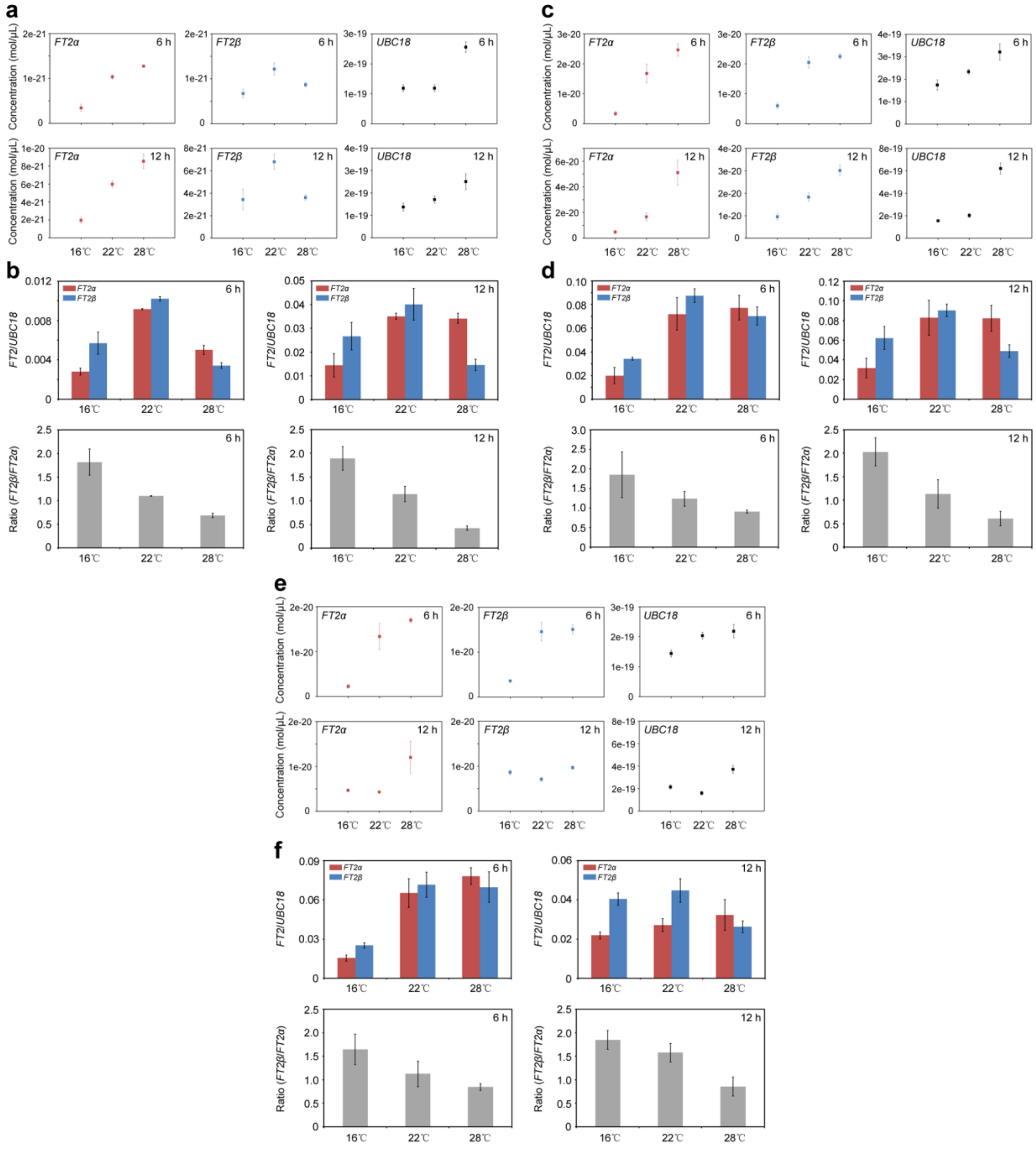

Supplemental Figure 3 AS of *FT2* in *B. distachyon* is affected by ambient temperature.

(a) Absolute value of *FT2α*, *FT2β* and *UBC18* in *B. distachyon* plants at 16℃, 22℃ and 28℃ for 6h and 12h, respectively, after shift from 22℃. Four-week old plants were shifted from 22℃ to 16℃, 22℃ and 28℃. y-axis means DNA concentration.

(b) Expression patterns of *FT2α* and *FT2β* and *FT2β/ FT2α* ratio in *B. distachyon* plants at 16℃, 22℃ and 28℃ for 6h and 12h after shift from 22℃. Four-week old plants were shifted from 22℃ to 16℃, 22℃ and 28℃.

(c)-(f) Two additional biological replicate results shown in (a) and (b).

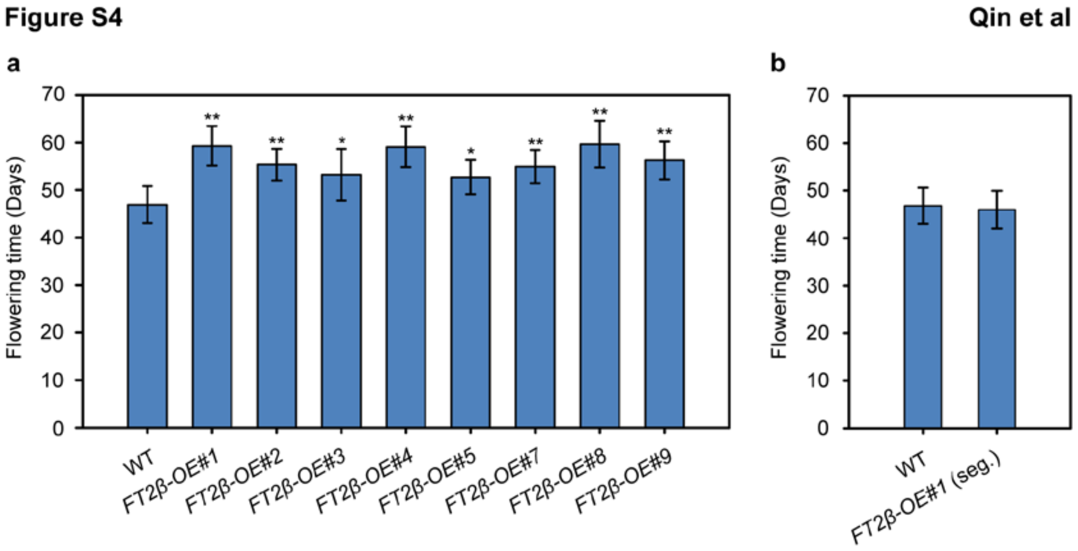

Supplemental Figure 4 *FT2β* overexpression represses flowering in *B. distachyon*.

(a) Flowering time of wild-type (WT) Bd21 and the independent lines of *FT2β-OE* transgenic plants. Error bars indicate SD (n=12).

(b) Flowering time of wild-type (WT) Bd21 and *FT2β-OE#1* null segregants.

Figure S5 **Qin et al**

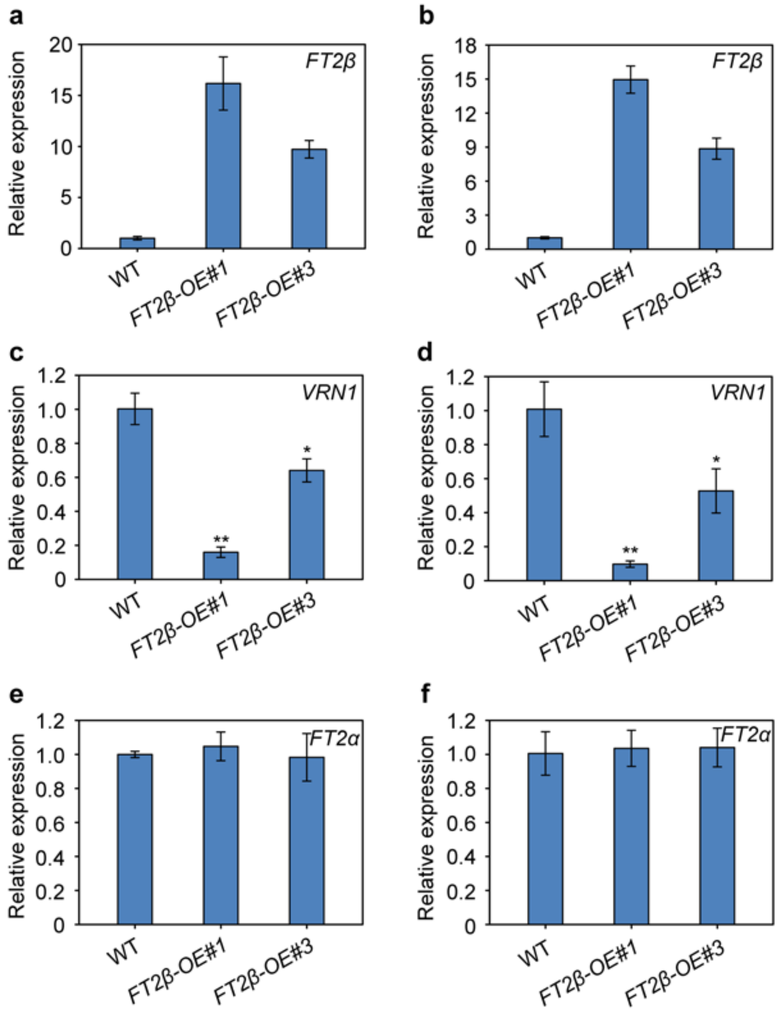

Supplemental Figure 5 qRT-PCR analyses of the flowering-related gene expression in wild-type Bd21 and *FT2β-OE* transgenic plants.

(a) and (b) Two additional biological replicate qRT-PCR results of *FT2β* expression in wild-type Bd21 and the indicated *FT2β-OE* transgenic plants. Related to Fig. 1d.

(c) and (d) Two additional biological replicate qRT-PCR results of downstream flowering gene *VRN1* expression in wild-type and the indicated *FT2β-OE* transgenic plants. Related to Fig. 1g.

(e) and (f) Two additional biological replicate qRT-PCR results of *FT2α* expression levels in wild-type and the indicated *FT2β-OE* transgenic plants. Related to Fig. 1h.

Figure S6 Qin et al

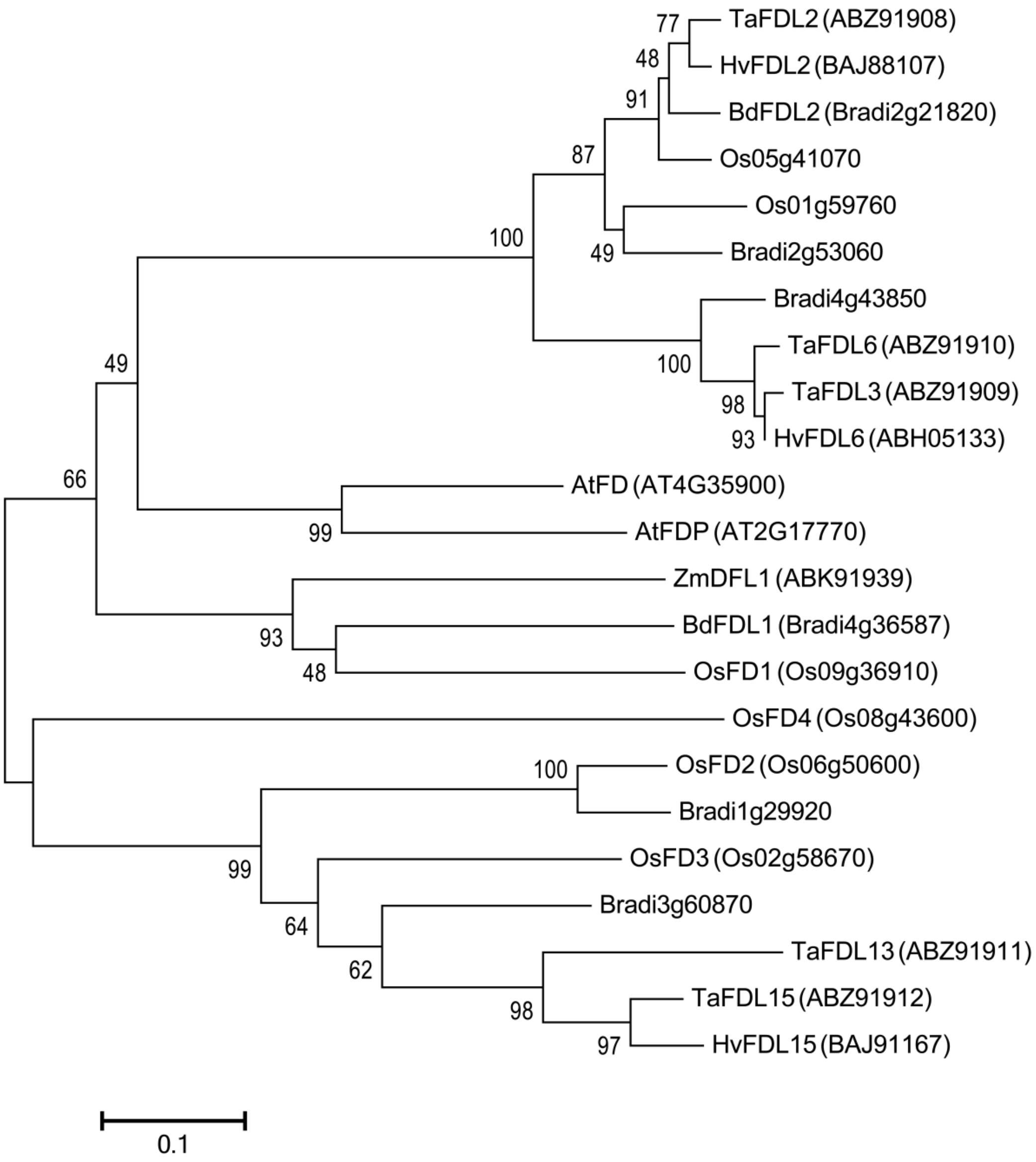

Supplemental Figure 6 A phylogenetic tree of FD-like proteins in *B. distachyon* and other plants.

Alignments of full-length FD-like protein sequences were produced by CLUSTALW and used for phylogenetic analysis. The midpoint-rooted phylogenetic tree was constructed by MEGA program using the Neighbor-Joining method. Bootstrap numbers shown at nodes are percentage of 1000 replicates.

Figure S7 Qin et al

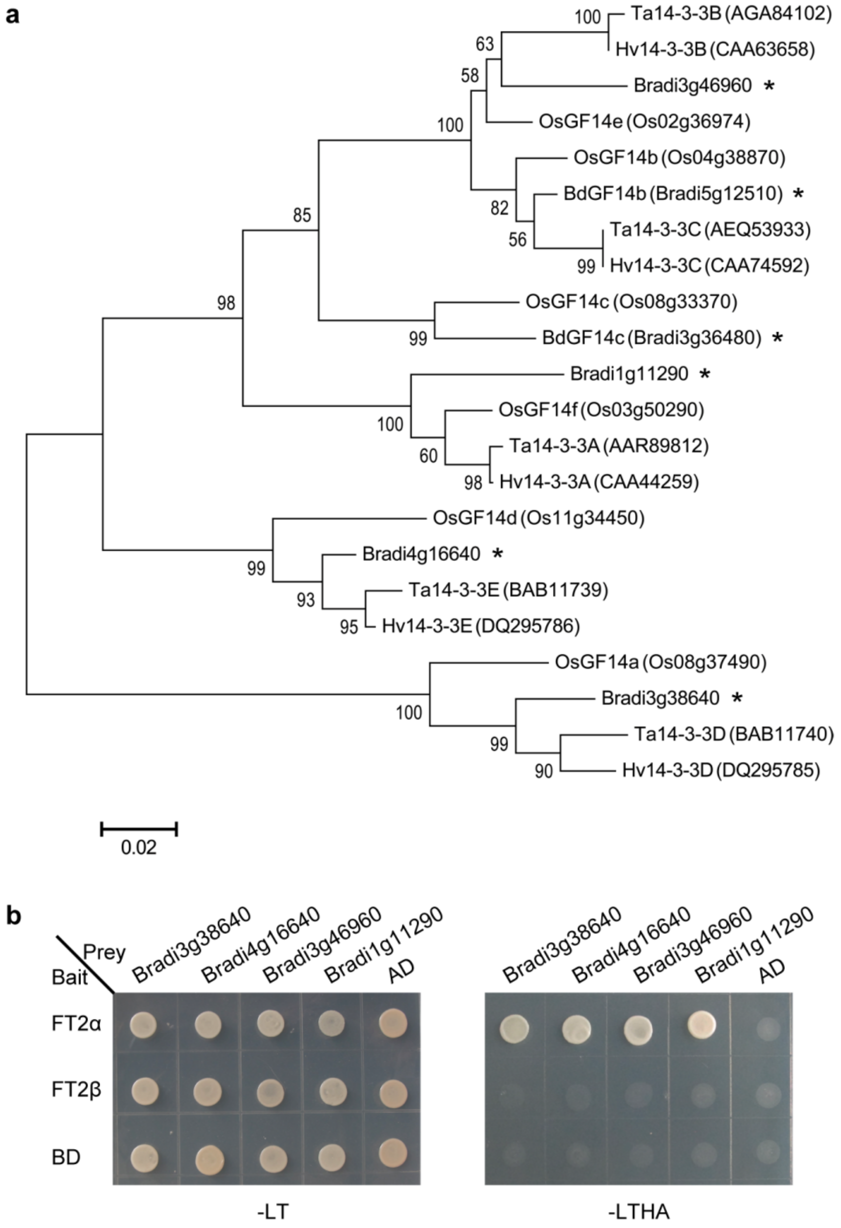

Supplemental Figure 7 FT2β cannot interact with 14-3-3 proteins

(a) A phylogenetic tree of 14-3-3 proteins in *B. distachyon* and other plants. Alignments of full-length 14-3-3 protein sequences were produced by CLUSTALW, and used for phylogenetic analysis. The midpoint-rooted phylogenetic tree was constructed by MEGA program using the Neighbor-Joining method. Bootstrap numbers shown at nodes are percentage of 1000 replicates. Star (*) indicates the 14-3-3 proteins that were selected to determine the interaction with FT2α and FT2β.

(b) Yeast two-hybrid analyses of FT2α and FT2β interactions with 14-3-3 proteins in different clades. Yeast cell growth on selective media without Leu, Trp, His and Ade (–LTHA) indicates positive interactions.

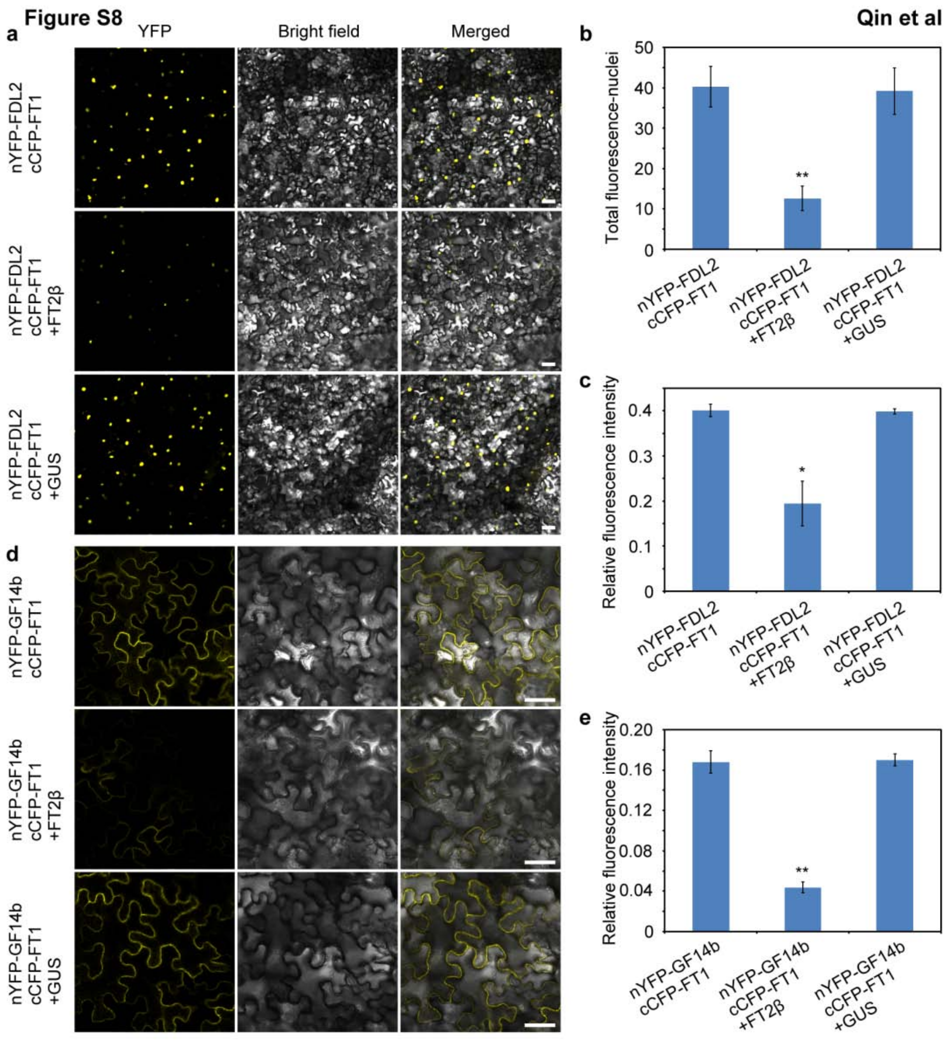

Supplemental Figure 8 Attenuation of binding capacity of FT1 with FD-like and 14-3-3 proteins by FT2β

(a) Representative photograph of fluorescence signals in BiFC assay for determination of FT2β effects on FT1 and FDL2 interactions in *N.benthamiana* leaves. GUS was used as a control in the absence of FT2β. Scale bar, 50 μm.

(b) Total fluorescence nuclei in BiFC assays used for analysis of FT2β inhibitory activities to FT1 and FDL2 interactions. Bars indicate standard error of the mean (Student's *t* test, **P <0.01).

(c) Relative fluorescence intensity in BiFC assays used for analysis of FT2β inhibition activity to FT1 and FDL2 interactions. Bars indicate standard error of the mean (Student's *t* test, *P <0.05).

(d) Representative photograph of fluorescence signals in BiFC assay for determination of FT2β effects on FT1 and 14-3-3 interactions in *N.benthamiana* leaves. GUS was used as a control in the absence of FT2β. Scale bar, 50 μm.

(e) Relative fluorescence intensity in BiFC assays used for analysis of FT2β inhibitory activities to FT1 and 14-3-3 interactions. Bars indicate standard error of the mean (Student's *t* test, **P <0.01)

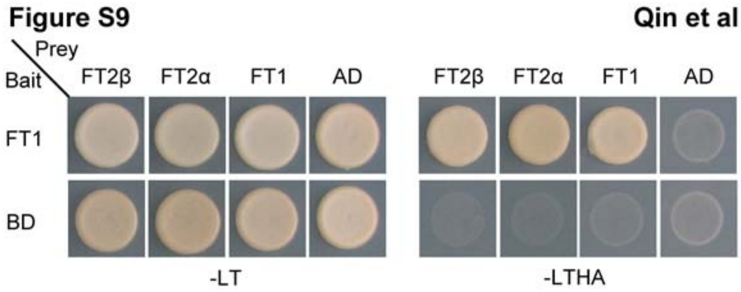

Supplemental Figure 9 FT1 can form homodimers, as well as interact with FT2α and FT2β to form herterodimers

Interaction analysis between FT1 and other indicated FTs was performed by yeast two-hybrid experiments. Yeast cell growth on selective media without Leu, Trp, His and Ade (–LTHA) represents positive interactions.

Figure S10 **Qin et al**

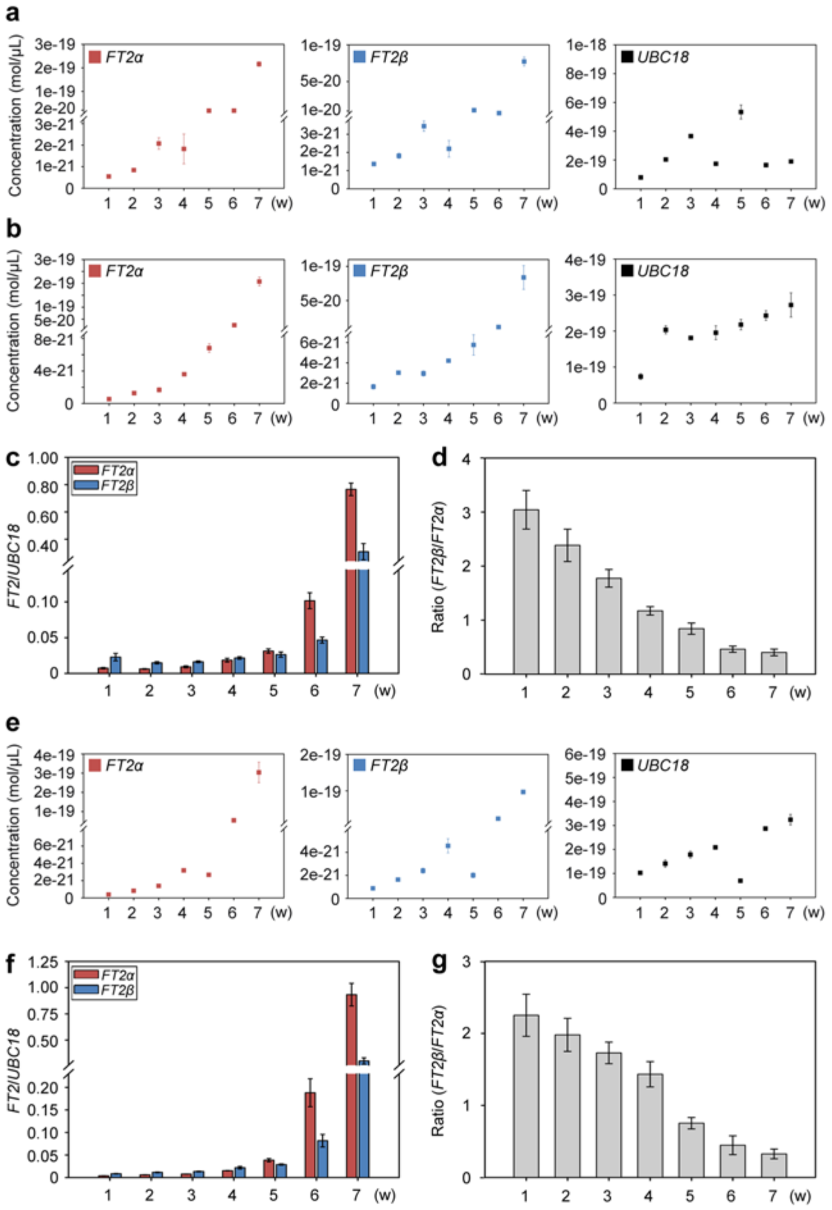

Supplemental Figure 10 age-dependent *FT2* AS

(a) Absolute quantification of *FT2α, FT2β* and *UBC18* transcripts in plants with different age. y-axis means DNA concentration. Error bars indicate SD. w, weeks. Related to Fig.5 a, b

(b) and **(e)** Two additional biological replicate results of absolute quantification of *FT2α, FT2β* and *UBC18* transcripts shown in (a).

(c) and **(f)** Two additional biological replicate results of *FT2α* and *FT2β* expression pattern in plants with different age. Related to Fig. 5a.

(d) and **(g)** Two additional biological replicate results of *FT2β/FT2α* ratio during plant development. Related to Fig. 5b.

Figure S11 **Qin et al**

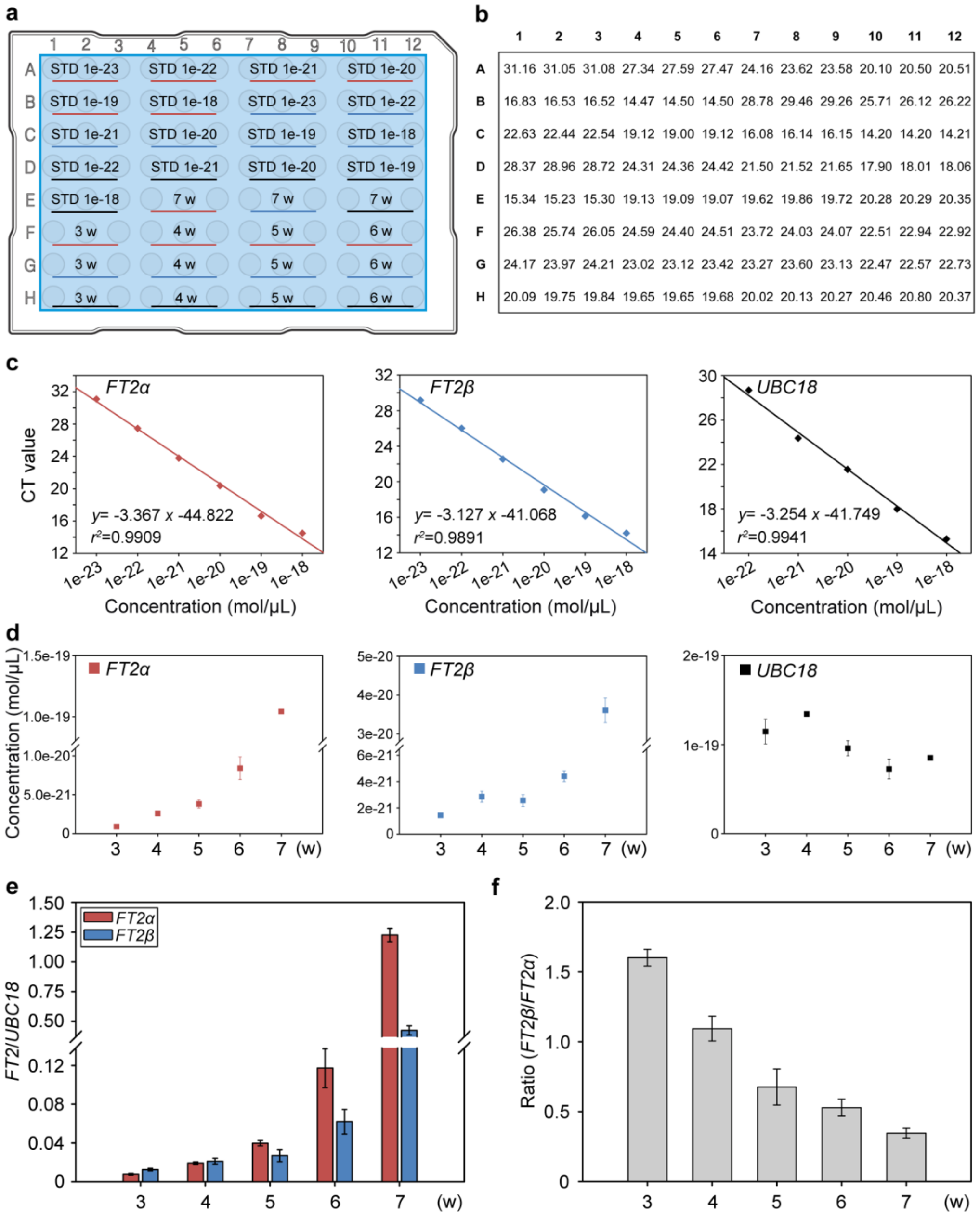

	1	2	3	4	5	6	7	8	9	10	11	12
A	31.16	31.05	31.08	27.34	27.59	27.47	24.16	23.62	23.58	20.10	20.50	20.51
B	16.83	16.53	16.52	14.47	14.50	14.50	28.78	29.46	29.26	25.71	26.12	26.22
C	22.63	22.44	22.54	19.12	19.00	19.12	16.08	16.14	16.15	14.20	14.20	14.21
D	28.37	28.96	28.72	24.31	24.36	24.42	21.50	21.52	21.65	17.90	18.01	18.06
E	15.34	15.23	15.30	19.13	19.09	19.07	19.62	19.86	19.72	20.28	20.29	20.35
F	26.38	25.74	26.05	24.59	24.40	24.51	23.72	24.03	24.07	22.51	22.94	22.92
G	24.17	23.97	24.21	23.02	23.12	23.42	23.27	23.60	23.13	22.47	22.57	22.73
H	20.09	19.75	19.84	19.65	19.65	19.68	20.02	20.13	20.27	20.46	20.80	20.37

Supplemental Figure 11 Absolute quantification of *FT2α*, *FT2β* and *UBC18* expressions in three- to seven-week-old *B. distachyon*

(a) The qRT-PCR reaction plate containing the samples of *B. distachyon* with different age and the indicated serial-dilution plasmids that are used to make standard curves of the used primers. The wells underlined with red, blue and black color represent the wells for *FT2α*, *FT2β* and *UBC18*, respectively. STD, Standards; w, week-old sample.

(b) The raw data of CT values of per well arranged on the plate shown in (a).

(c) Standard curve for quantification of *FT2α*, *FT2β* and *UBC18* transcripts.

(d) Absolute value of *FT2α*, *FT2β* and *UBC18* in three- to seven-week-old *B. distachyon* plants.

(e) Expression levels of *FT2α* and *FT2β* in *B. distachyon* with the indicated age.

(f) *FT2β/FT2α* ratios in three- to seven-week-old *B. distachyon* plants.

Figure S12 **Qin et al**

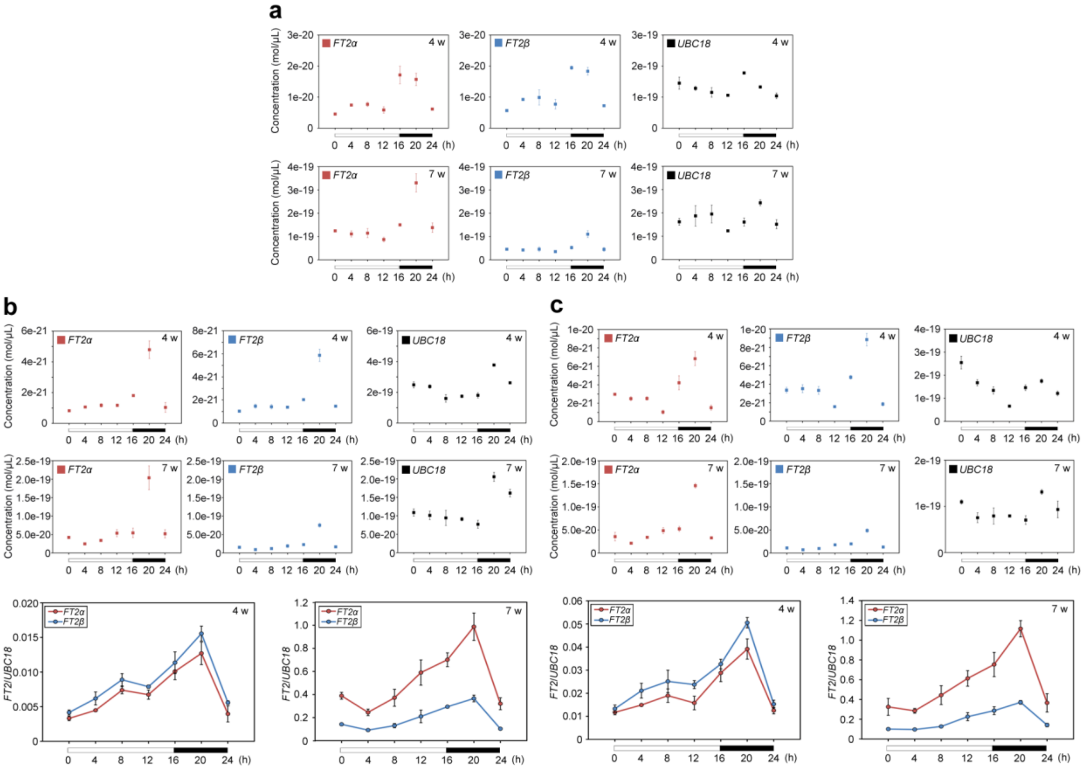

Supplemental Figure 12 Diurnal expression analysis of *FT2α* and *FT2β* in 24-h period in four- and seven-week-old *B. distachyon*

(a) Absolute value of *FT2α*, *FT2β* and *UBC18* in 24-h period in four- and seven-week-old *B. distachyon* plants. The white and black bars along the horizontal axes represent light and dark periods, respectively. y-axis means DNA concentration. The numbers below the horizontal axes indicate the time in hours. Each qRT-PCR analysis were performed three biological replicates with similar results. The point represents the average of three technical replicates in a representative biological experiment. Error bars indicate SD. h, hours. w, weeks. Related to Fig.5c,d

(b) and **(c)** Two additional quantification results of *FT2α*, *FT2β* and *UBC18* expressions in 24-h period in four- and seven-week-old *B. distachyon* plants. Related to Fig.5c,d and Supplemental Figure 12a.

a

```
ATGCCGCGGGGGGATCCGCTGGTGGTGGGGCGGGTGATAGGCGACGTGGTGGACCCGTTCGTGCGGCGCGTGTCGCTGCGGGTCGGG
TACGCGTCCAGGGACGTGGCCAACGGCTGCGAGCTCCGCCCCTCCGCCATCGCCGACCCGCCCCGCGTCGAGGTCGGCGGCCCCGACA
TGCGCACCTTCTACACGCTGGTGATGGTGGATCCGGACGCGCCCAGCCCCAGCGATCCCAGCCTTAGGGAGTACTTGCACTGGCTGGTCA
CCGACATCCCGGCTACGACAGGAGTATCTTTTGGGACTGAGGTGGTGTGCTACGAGAGCCCGCGGCCGGTGCTCGGAATTCACCGGCTG
GTGTTCCTTCTATTCCAGCAGCTCGGCCGGCAGACGGTGTACGCTCCGGGTTGGCGGCAGAACTTCAGCACCCGCGACTTCGCCGAGCTG
TACAACCTCGGCTTGCCGGTCGCCGCTGTTTACTTCAACTGCCAAAGGGAGTCTGGAACTGGAGGGAGAAGAATGTGA
```

b *BdMIR172a*

```
A--      CACGCAC---       -   C     A            C  C          A      -    ---|    AG      AAUU
   GGCGG            AGUCG  GCG  UUGC  GGUGCAGCA  CA  CAAGAUUC  CAUCG  GGUU     CUCC  UCGU      \
   CUGCU            UCAGC  CGC  AACG  CUACGUCGU  GU  GUUCUAAG  GUAGU  CCAA     GAGG  AGCA      A
UAA      AUACUACUCA       U   C     G            A  A          A      A    CAU^    --      AUUA
```

Artificial MIRFT2α

```
A--      CACGCAC---       -   C     A            C  A          A      -    ---|    AG      AAUU
   GGCGG            AGUCG  GCG  UUGC  GCGUGGCCA  CG  CUGCGAGC  CAUCG  GGUU     CUCC  UCGU      \
   CUGCU            UCAGC  CGC  AACG  CGCACCGGU  GC  GACGCUCG  GUAGU  CCAA     GAGG  AGCA      A
UAA      AUACUACUCA       U   C     G            U  C          A      A    CAU^    --      AUUA
```

Artificial MIRFT2β

```
A--      CACGCAC---       -   C     A            A  A          A      -    ---|    AG      AAUU
   GGCGG            AGUCG  GCG  UUGC  GGUUCGUGC  GC  CGUCGAGG  CAUCG  GGUU     CUCC  UCGU      \
   CUGCU            UCAGC  CGC  AACG  CCAAGCACG  CG  GCAGCUCC  GUAGU  CCAA     GAGG  AGCA      A
UAA      AUACUACUCA       U   C     G            C  C          A      A    CAU^    --      AUUA
```

Supplemental Figure 13 The strategy of artificial miRNA design to silence *FT2* splicing variants

(a) Full-length cDNA sequence of *FT2*. Red color-marked nucleotides indicate the sequences that are missing in *FT2β*. Nucleotides underlined by red and green color indicate the amiRFT2α and amiRFT2β target site, respectively.

(b) Secondary structures of precursor of miR172a, amiRFT2α and amiRFT2β in *B. distachyon*. Red color-marked nucleotides indicate the mature miRNA.

Figure S14 Qin et al

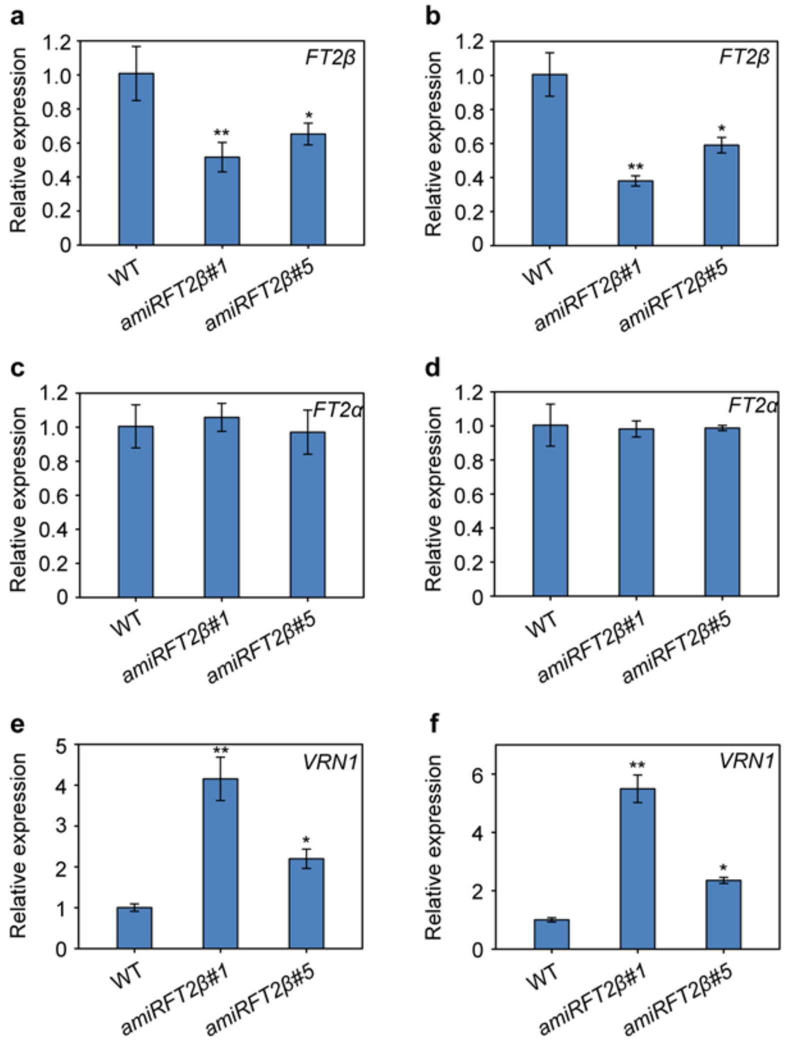

Supplemental Figure 14 qRT-PCR analysis of the flowering-related gene expression in wild-type Bd21-3 and *amiRFT2β* transgenic plants.

(a) and (b) Two additional biological replicate qRT-PCR results of *FT2β* expression in wild-type Bd21-3 and the indicated *amiRFT2β* transgenic plants. Related to Fig. 6c.

(c) and (d) Two additional biological replicate qRT-PCR results of *FT2α* expression in wild-type and the indicated *FT2β-OE* transgenic plants. Related to Fig. 6d.

(e) and (f) Two additional biological replicate qRT-PCR results of *VRN1* expression levels in wild-type and the indicated *FT2β-OE* transgenic plants. Related to Fig. 6g.

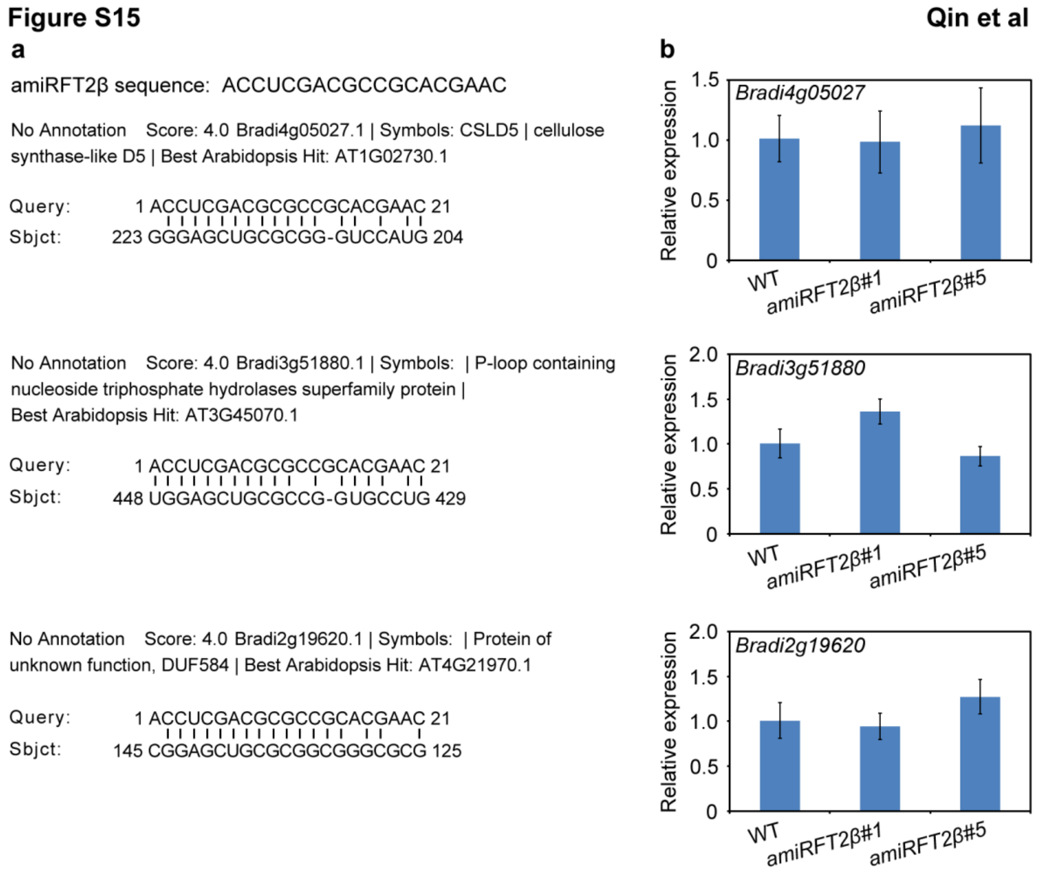

Supplemental Figure 15 Early flowering of *amiRFT2β* transgenic plants is not a result of its unspecific potential targets

(a) Sequence alignment of of amiRFT2β and its potential targets. The potential targets of amiRFT2β were predicted by bioinformatics analysis. Firstly, an initial pool of predicted targets were created, with at most four unpaired nucleotides (including G:U pairs, mismatches, and single-nucleotide bulges) and a single-nucleotide bulge allowed between a miRNA and its targets. A mis-pair scoring system was then applied to these initial targets. Mismatches and single-nucleotide bulges were each scored as 1, and G:U pairs were each scored as 0.5. The scores were doubled if mismatches, G:U pairs, and bulges were located at positions at 2 to 13 as counted from the 5'-end of a miRNA. Genes with a mis-pair score ≦4 were selected as putative miRNA targets.

(b) qRT-PCR analysis of amiRFT2β potential target gene expressions in wild-type and *amiRFT2β* transgenic plants.

Figure S16 **Qin et al**

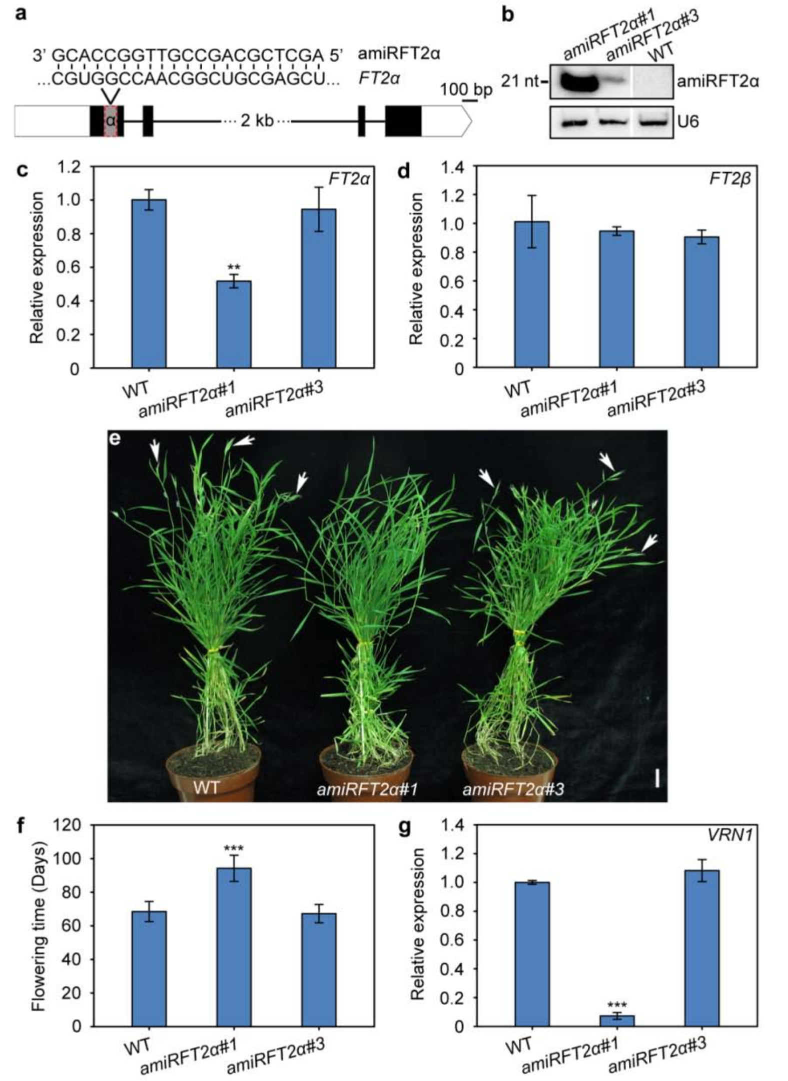

Supplemental Figure 16 Interruption of FT2α activity by artificial miRNA cannot lead to early flowering

(a) Schematic diagram of *amiRFT2α* targeting the designed sequences for *FT2α*.

(b) Northern blot analysis of artificial miRNA expression in *amiRFT2α* transgenic plants. U6 was used as a loading control for RNA gel blot.

(c) qRT-PCR analysis of *FT2α* expression in wild-type and the indicated *amiRFT2α* transgenic plants. *UBC18* was used as an internal control for normalization of qRT-PCR results.

(d) qRT-PCR analysis of *FT2β* expression in wild-type and the indicated *amiRFT2α* transgenic plants.

(e) Representative photograph showing flowering phenotypes in *amiRFT2α* transgenic and wild-type (Bd21-3) plants. White arrows point to spikes. Scale bar, 4cm.

(f) Flowering time of wild-type Bd21-3 and the indicated two lines of *amiRFT2α* transgenic plants. Error bars indicate SD (n=12).

(g) qRT-PCR analysis of flowering downstream gene *VRN1* expression in wild-type and the indicated *amiRFT2α* transgenic plants.

Figure S17 **Qin et al**

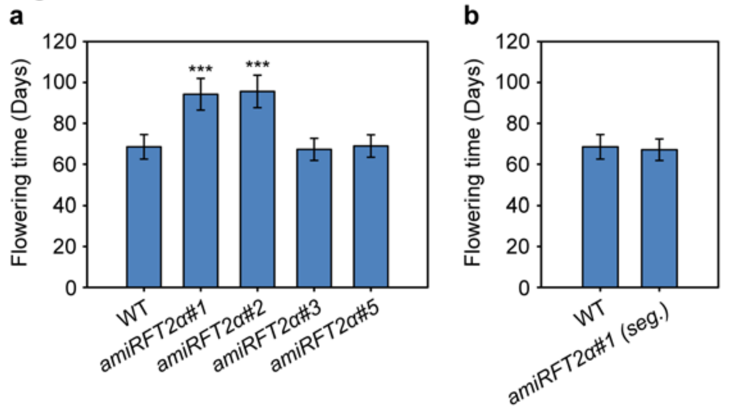

Supplemental Figure 17 Down-regulation of *FT2α* cannot promote flowering in *B. distachyon*.

(a) Flowering time of wild-type (WT) Bd21-3 and the independent *amiRFT2α* transgenic plants. Error bars indicate SD (n=12).

(b) Flowering time of wild-type (WT) Bd21-3 and *amiRFT2α#1* null segregants of plants.

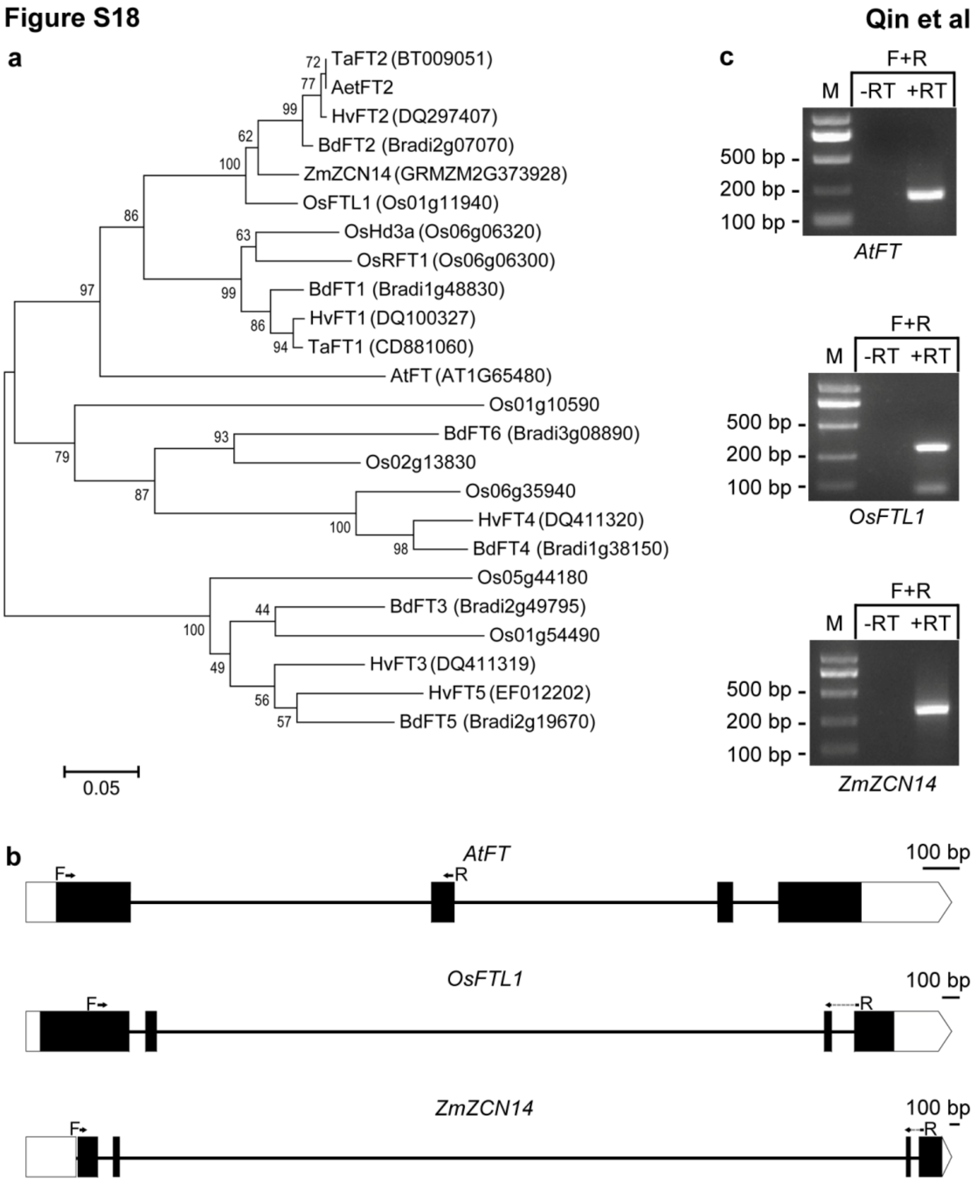

Supplemental Figure 18 Endogenous cue-dependent *FT2* AS is not present in all Poaceae plants

(a) A phylogenetic tree of FT proteins in temperate grasses and other plants. Alignments sequences were produced by CLUSTALW, and used for phylogenetic analysis. The midpoint-rooted phylogenetic tree was constructed by MEGA program using the Neighbor-Joining method. Bootstrap numbers shown at nodes are percentage of 1000 replicates.

(b) Schematic gene structures of *FT* in *A.thaliana*, rice and maize. White boxes indicate untranslated regions and black boxes indicate exons. Primers used for PCR amplification are marked.

(c) RT–PCR analysis of *FT* transcripts in *A.thaliana*, rice and maize. M, marker. bp, base pairs.

Figure S19 **Qin et al**

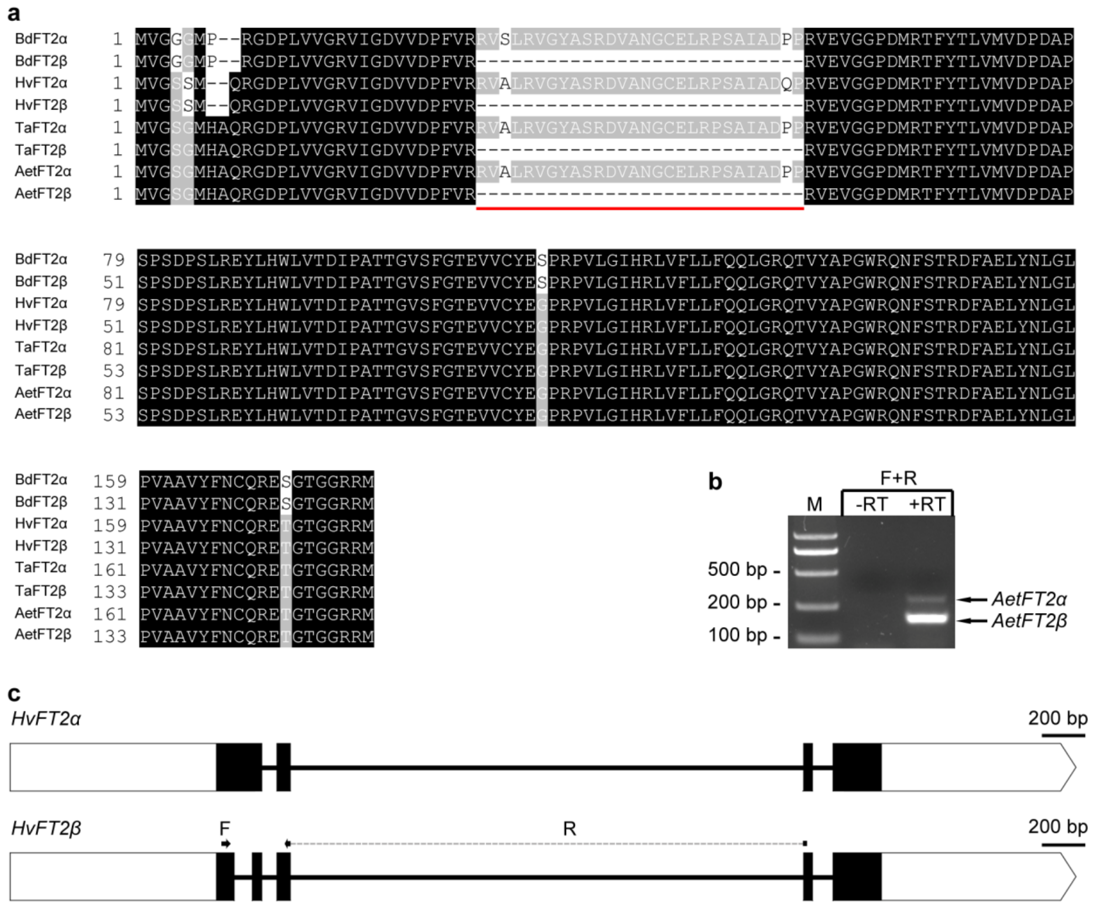

Supplemental Figure 19 Endogenous cue-dependent *FT2* AS is conserved in temperate grasses

(a) Same protein region of FT2 is missing in FT2β in *B. distachyon*, barley, wheat and *Ae. tauschii*. The missing amino acids are underlined.

(b) Detection of *FT2* AS transcripts by RT–PCR in *Ae.tauschii*. Primers designed for simultaneous amplification of *FT2α* and *FT2β* in a single PCR reaction were used. M, marker.

(c) Schematic gene structures of *FT2* splicing variants in barley. White boxes indicate untranslated regions and black boxes indicate exons. Primers used for *FT2α* and *FT2β* simultaneous amplification are marked. bp, base pairs.

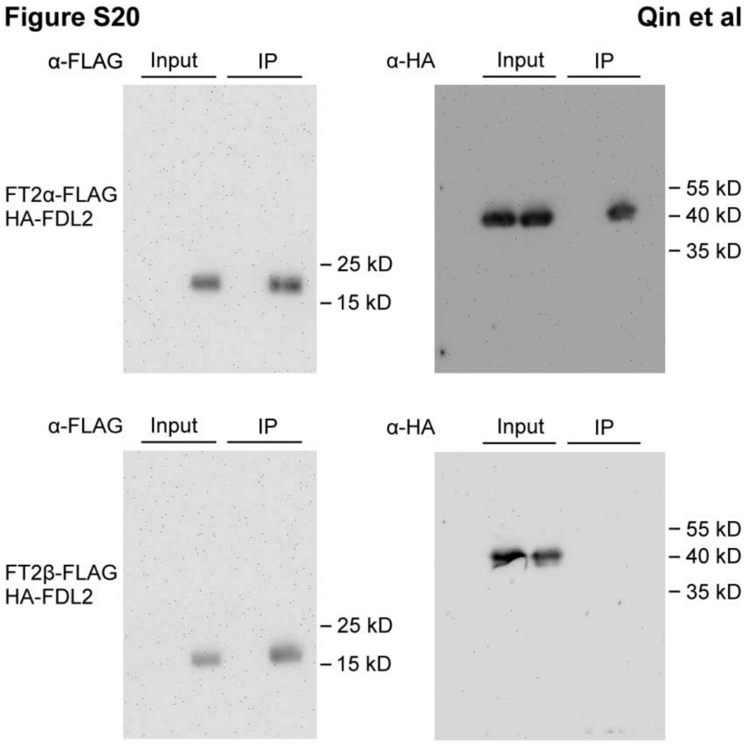

Supplemental Figure 20 Full scan picture of western blot results from FT2α and FT2β with FDL2 related in Fig. 2c

Supplemental Table 1.Primers used for this research

Primer Name	Primer Sequence(5′ to 3′)
**Gene cloning and vector construction**	
FT2β-1390-F	TTACTTCTGCACTAGGTACCATGCCGCGGGGGATCCGCT
FT2β-1390-R	GAATTCCCGGGGATCCTCACATTCTTCTCCCTCCAGTTCC
miR172a-F	TGTACATGTTCGATCCCCACC
miR172a-R	CGGTCATCATCATCAGAAGAAG
amiR-1390-F	TTACTTCTGCACTAGGTACCTGTACATGTTCGATCCCCACC
amiRFT2β-1R	TGTCCTCGACGTGCTGCACGAACCTGCAAGCGCCGACTGT GCG
amiRFT2β-2F	TCGTGCAGCACGTCGAGGACATCGGGTTCTCCAGTCGTAA TTAATTAACGAGGAG
amiRFT2β-2R	GTTCGTGCGGCGCGTCGAGGTCATCATGGTTGTACTCCTC GTTAATTAATTACGA
amiRFT2β-3F	ACCTCGACGCGCCGCACGAACCGGCAACCGCTCGACTACT CA
amiR-1390-R	GAATTCCCGGGGATCC CGGTCATCATCATCAGAAGAAG
amiRFT2α-1R	CGACTGGAGAACCCGATGTGCTCGCAGTCGGTGGCCACGC TGCAAGCGCCGACTGTGCG
amiRFT2α-2F	GCTTGCAGCGTGGCCACCGACTGCGAGCACATCGGGTTCT CCAGTCGTAATTAATTAAC
amiRFT2α-2R	GTAGTCGAGCGGTTGCCGCGTGGCCAACGGCTGCGAGCT CATCATGGTTGTACTCCTCG
amiRFT2α-3F	TGATGAGCTCGCAGCCGTTGGCCACGCGGCAACCGCTCGA CTACTCATCATATCGTCAA
FT2-BD-F	CATGGAGGCCGAATTCATGCCGCGGGGGATCCGCT
FT2-BD-R	GCAGGTCGACGGATCCCATTCTTCTCCCTCCAGTTCC
FDL2-AD-F	GGAGGCCAGTGAATTCATGATTCAGGCAATGTCGTCG
FDL2-AD-R	CGAGCTCGATGGATCCAACAGGGGAAGAGCTTGTTCT
GF14b-AD-F	GGAGGCCAGTGAATTCATGTCGGCACCTGCGGAGCTT
GF14b-AD-R	CGAGCTCGATGGATCCCTGCCCATCACCAGAGTCACC
GF14c-AD-F	GGAGGCCAGTGAATTCATGTCGCGGGAGGACAATGTT
GF14c-AD-R	CGAGCTCGATGGATCCCTGGCCCTCGCCAGCTTCACCTT
Bradi1g11290-AD-F	GGAGGCCAGTGAATTCATGTCTACTGCTGAGGCAACCCGT
Bradi1g11290-AD-R	CGAGCTCGATGGATCCGCACTGTAGTTTTGTACACCCCCAC
Bradi3g46960-AD-F	GGAGGCCAGTGAATTCATGGCACAGACTGTGGAGCTTTCC
Bradi3g46960-AD-R	CGAGCTCGATGGATCCCTGTCCGTCTCCAGATTCTCCCTTG

Bradi4g16640-AD-F	GGAGGCCAGTGAATTCATGTCGCCGGCGGAGCCGAC
Bradi4g16640-AD-R	CGAGCTCGATGGATCCCTGTCCATCTCCAGATTCTTTTG
Bradi3g38640-AD-F	GGAGGCCAGTGAATTCATGGCTGCAGCGGCGGCGGC
Bradi3g38640-AD-R	CGAGCTCGATGGATCCGTGCTCATCATCCTCAGGCTTG
FT1-BD-F	CATGGAGGCCGAATTCATGGCCGGGAGGGACAGG
FT1-BD-R	GCAGGTCGACGGATCCGGGGTACATCCTCCTGCCG
FT2-BiFC-F	CAAAAAAGCAGGCTTCATGCCGCGGGGGGATCCGCT
FT2-BiFC-R	CAAGAAAGCTGGGTCCATTCTTCTCCCTCCAGTTCC
FDL2-BiFC-F	CAAAAAAGCAGGCTTCATGATTCAGGCAATGTCGTCG
FDL2-BiFC-R	CAAGAAAGCTGGGTCAACAGGGGAAGAGCTTGTTCT
GF14b-BiFC-F	CAAAAAAGCAGGCTTCATGTCGGCACCTGCGGAGCTT
GF14b-BiFC-R	CAAGAAAGCTGGGTCCTGCCCATCACCAGAGTCACC
FT1-BiFC-F	CAAAAAAGCAGGCTTCATGGCCGGGAGGGACAGGGAC
FT1-BiFC-R	CAAGAAAGCTGGGTCGGGGTACATCCTCCTGCCGCC
FT2-Flag-F	GGGTACCCGGGGATCCATGCCGCGGGGGGATCCGCT
FT2-Flag-R	CCATGTCGACTCTAGACATTCTTCTCCCTCCAGTTCC
**qRT-PCR**	
FT2α-F	TGCGAGCTCCGCCCCTCCGCCAT
FT2β-F	GTTCGTGCGGCGCGTCGAGGTCG
FT2-R	TGGCAGTTGAAGTAAACAGCG
VRN1-F	GCTCTGCAGAAGGAACTTGTGG
VRN1-R	CGCTGCTGGGCGATTACTGAT
UBC18-F	GGAGGCACCTCAGGTCATTT
UBC18-R	ATAGCGGTCATTGTCTTGCG
Bradi4g05027-F	TGAGGCCTACTTCGGGCAGAAG
Bradi4g05027-R	CCCTCACCTTGAACTCGTCGTACTC
Bradi3g51880-F	ACCACATGGCCGAGTACTGGAAGGA
Bradi3g51880-R	TGAAAGGGACGCCGAGGAACTG
Bradi2g19620-F	ATCGGGAGCTCGAGGAAGCCGAAG
Bradi2g19620-R	ATGGGCCTGCTGCTTGCCGATC
**RT-PCR**	
BdFT1-F	ATGGCCGGGAGGGACAGGGAC
BdFT1-R	TCAGGGGTACATCCTCCTGCCG
BdFT1-F1	GTTGGCAGGGTTGTGGGGGAC
BdFT1-R1	GGCTCTCGTAGCACATCACCTCCTG
BdFT2-F	TGATAGGCGACGTGGTGGACCCGTT
BdFT2-R	AGCCGGTGAATTCCGAGCAC
HvFT2-F	CGACGTGGTGGACCCGTTCGT

HvFT2-R	GGATGTCGGTGACCAGCCAGTG
HvActin-F	GCCGTGCTTTCCCTCTATG
HvActin-R	GCTTCTCCTTGATGTCCCTTA
TaFT2-F	GCGTGATCGGCGACGTGGTGGA
TaFT2-R	TCGGTGACCAGCCAGTGCAAGTAC
TaGAPDH-F	GTTGAGGGTTTGATGACCAC
TaGAPDH-R	TCAGACTCCTCCTTGATAGC
AtFT-F	GTAAGCAGAGTTGTTGGAGACG
AtFT-R	GATATTCTCGGAGGTGAGGGTTGC
OsFTL1-F	ACGTGGTGGACCCGTTCGTGA
OsFTL1-R	TAGCACACCACCTCTGTCCCAAAAG
ZmZCN14-F	ATCCGCTGGTGGTGGGCCGCATCAT
ZmZCN14-R	ACACGACCTCGGTCCCAAAAGATAC
AetFT2-F	CGACGTGGTGGACCCGTTCGT
AetFT2-R	CCTCGGTCCCAAAAGACACTCCT
Hyg-F	GCAAGACCTGCCTGAAACCGA
Hyg-R	CTATCGGCGAGTACTTCTACACAG
**Probes for RNA gel blot**	
amiRFT2β	GTTCGTGCGGCGCGTCGAGGT
amiRFT2α	CGTGGCCAACGGCTGCGAGCT
U6	TGTATCGTTCCAATTTTATCGGATGT
